# The Use of Adjunctive Steroids in Central Nervous Infections

**DOI:** 10.3389/fcimb.2020.592017

**Published:** 2020-11-23

**Authors:** Shalini Gundamraj, Rodrigo Hasbun

**Affiliations:** ^1^ Cornell University, Ithaca, NY, United States; ^2^ Department of Internal Medicine, UT Health McGovern Medical School, Houston, TX, United States

**Keywords:** meningitis, encephalitis, steroids, central nervous system infections, brain abscess, cysticercosis

## Abstract

Central nervous system (CNS) infections continue to be associated with significant neurological morbidity and mortality despite various existing therapies. Adjunctive steroid therapy has been employed clinically to reduce inflammation in the treatment of CNS infections across various causative pathogens. Steroid therapy can potentially improve clinical outcomes including reducing mortality rates, provide no significant benefit, or cause worsened outcomes, based on the causative agent of infection. The data on benefits or harms of adjunctive steroid therapy is not consistent in outcome or density through CNS infections, and varies based on the disease diagnosis and pathogen. We summarize the existing literature on the effects of adjunctive steroid therapy on outcome for a number of CNS infections, including bacterial meningitis, herpes simplex virus, West Nile virus, tuberculosis meningitis, cryptococcal meningitis, *Angiostrongylus cantonensis*, neurocysticercosis, autoimmune encephalitis, toxoplasmosis, and bacterial brain abscess. We describe that while steroid therapy is beneficial and supported in pathogens such as pneumococcal meningitis and tuberculosis, for other diseases, like *Listeria monocytogenes* and *Cryptococcus neoformans* they are associated with worse outcomes. We highlight areas of consistent and proven findings and those which need more evidence for supported beneficial clinical use of adjunctive steroid therapy.

## Introduction

Central nervous system (CNS) infections involve the brain, spine, and associated membranes, and are linked to significant neurological morbidity and mortality, with long term consequences in survivors that affect the quality of life and activity of daily living (ADLS) (Erdem et al., 2017; Sulaiman et al., 2017). CNS infection broadly can be categorized as encephalitis, meningitis, or intracranial suppurative complications (e.g., brain abscess), with a broad range of causal organisms and clinical presentations. CNS infections can either originate by hematogenous spread (e.g., bacteremia, viremia), by retrograde neuronal invasion (e.g., viral infection through axonal transport such as rabies, *Naegleria fowleri*) or by contiguous spread of microorganisms (e.g., post cranial trauma or surgery, implementation of medical hardware into the brain or spine, or by parameningeal spread from a focus such as sinusitis or mastoiditis) (Archibald and Quisling, 2013; Koyuncu et al., 2013). Various bacteria, fungi, viruses, and parasites can be the source of CNS infection, which often presents nonspecifically with headache, fever, altered mental status, and behavioral changes (Dorsett and Liang, 2016). Precision in diagnosis, however, is essential to treatment, which varies across CNS infection. Despite the use of antimicrobial therapies neurological morbidity and mortality remains high for certain pathogens such as *Streptococcus pneumoniae* (Steel et al., 2013). Of all the adjunctive therapies that have been evaluated to date only adjunctive steroids have shown benefit in some CNS infections. Adjunctive steroids can ameliorate the host’s inflammatory response to the infection that account for the neurological morbidity associated with the CNS infection. In contrast, in some CNS infections, the use of steroids has no clear beneficial effects or is detrimental with worse outcomes (Fitch and van de Beek, 2008). In this review, we will review the available data for the use of adjunctive steroids in the most common CNS infections and make clear recommendations for its use or not.

## Pathophysiology for Use of Adjunctive Steroids in Meningitis

Meningitis is characterized by inflammation of the subarachnoid space (space between two membranes (i.e., meninges) that surrounds the brain and spinal cord). Meningitis can be caused by bacteria, viruses, fungus, amebic, parasites, mycobacteria or due to noninfectious causes. Patients typically have abnormalities in the cerebrospinal fluid (CSF) such as elevated white blood cells (WBC), elevated protein (due to alterations in the blood brain barrier), and low glucose in some cases. The presentation of meningitis can be classified as acute (symptoms for less than 5 days), subacute (symptoms 6-30 days), or chronic (symptoms for more than 30 days) (Sulaiman et al., 2017). Presentation clinically varies according to the age of the patient, the causative agents, and the presence of underlying conditions (Hasbun et al., 2018). Once bacteria invades the meninges and disrupts the blood-brain barrier, it rapidly replicates in the subarachnoid space (van de Beek et al., 2016a). In the physiological response to infection, blood vessels become leaky allowing fluid, leukocytes, and other larger particles involved in fighting infection to enter the meninges and the brain (Dando et al., 2014). This process causes severe inflammation, which has been shown to increase the adverse outcomes of the infection. Steroid therapy has been employed to reduce the inflammatory response commonly exhibited in meningitis, although its effects vary by the study population studied and by the pathogen (Hoffman and Weber, 2009). Corticosteroid therapy can reduce inflammation by suppressing multiple activated inflammatory genes (encoding pro-inflammatory cytokines. and chemokines, inflammation mediators, adhesion molecules, inflammatory enzymes, etc.) through primarily reversing histone acetylation, by decreasing cell activation and recruitment and stabilizing lysosomes, by temporarily alleviating the leaky blood brain barrier, and by interacting with DNA recognition sites to activate the transcription of anti-inflammatory genes in higher concentrations in humans (Coyle, 1999; Barnes, 2006). In animal models of bacterial meningitis, studies have shown outcomes to worsen based on the degree of inflammation (Scheld et al., 1980; Tauber et al., 1985; Brouwer et al., 2018). This inflammatory response can be reduced in the CSF by the administration of corticosteroid therapy, resulting in improved outcomes for meningitis in animal models (Scheld et al., 1980; Tauber et al., 1985). Thus, the use of corticosteroids has been investigated and employed in meningitis as adjuvant therapy over the past 30 years but must be further investigated in some infections.

## Bacterial Meningitis by Pathogen

Bacterial meningitis is a severe infection and inflammation of the meninges, the membrane surrounding the brain and spine, due to bacterial invasion into the subarachnoid space (Hoffman and Weber, 2009). The condition is associated with high mortality and neurological morbidity rates, with the most common causal pathogens including *S. pneumoniae (i.e., pneumococcal)*, *Neisseria meningitides (i.e., meningococcal)*, *Haemophilus influenzae*, and *Listeria monocytogenes* (Hoffman and Weber, 2009). Due to the development and implementation of vaccines against *H. influenzae* type b, *S. pneumoniae*, and *N. meningitidis* the incidence of bacterial meningitis has been reduced to 1–2/100,000 in children (Hasbun et al., 2018). In infants from 4–6 weeks, the dominating etiologies are *Streptococcus agalactiae, Escherichia coli*, other Enterobacteriaceae, and *L. monocytogenes.* In children older than 6 weeks up to adults of age 50 years, *S. pneumoniae*, *N. meningitidis*, and *H. influenzae* are the dominating etiologies of infection. In both adults over the age of 50 years and immunocompromised individuals of all ages, *S. pneumoniae*, *N. meningitidis*, *H. influenzae*, and *L. monocytogenes* prevail (Hasbun et al., 2018).

### *S. pneumoniae*

*S pneumoniae* is the leading cause of bacterial meningitis in the USA (Castelblanco et al., 2014). A review in 2018 of corticosteroid treatment effects in 25 trials across 2,511 children and 1,517 adults showed there was no significant change in mortality found overall with steroid therapy, although an effective mortality reduction was seen for *S. pneumoniae* meningitis from 36% to 29.9%. The reduction in mortality for *S, pneumoniae* meningitis in 16 high-income countries was attributed to the use of adjunctive steroid therapy as compared to patients who did not receive steroid therapy. Patient outcome was not significantly different with the use of adjunctive steroids in low-income countries, which made up 9 of the 25 analyzed studies. Dexamethasone was the specific corticosteroid administered as adjunctive therapy in 22 of the trials, while hydrocortisone and prednisone were administered in the remaining 3 studies (Brouwer et al., 2018). No significant adverse effects were reported in the clinical studies by the use of adjunctive steroid therapy. The lack of effect of corticosteroid use in low-income countries, contrasted with significant reductions in mortality with corticosteroid adjunctive treatment for *S. pneumoniae* in high-income countries, is most likely due to the delay in patient presentation and treatment in low-income countries after inflammation in the CNS has already begun and progressed (van de Beek et al., 2016b). A 2002 randomized control trial on the effects of dexamethasone treatment in adults with meningitis similarly found a reduction in mortality and adverse outcomes, seizure incidence, and cardiorespiratory failure in contrast with the placebo group in pneumococcal meningitis (de Gans and van de Beek, 2002) (see **Table 1**). Dexamethasone corticosteroid adjunctive therapy has been shown to reduce mortality in pneumococcal meningitis and is a recommended therapy by the Infectious Diseases Society of America (IDSA), European and United Kingdom guidelines (Tunkel et al., 2004; NICE UK, 2010; McGill et al., 2016; van de Beek et al., 2016b). The implementation of dexamethasone treatment in the Netherlands has shown a decrease of 10% in the mortality rate of pneumococcal meningitis (Brouwer et al., 2010). A study of 26,429 adults from 2011–2014 with a discharge diagnosis of meningitis or encephalitis showed that only patients with pneumococcal meningitis had a significantly reduced mortality with the use of adjunctive intravenous steroids. The study had 572 patients with pneumococcal meningitis and showed a decrease in hospital mortality rate with the use of steroids [(6.67% with and 12.5% without steroids, (P=0.0245)] (Hasbun et al., 2017) (see **Table 1**). Recently, delayed cerebral injury (DCI) in patients with bacterial meningitis has been described in up to 4% of patients and has a possible association with the administration of adjunctive steroid (Gallegos et al., 2018). Pathological studies suggest a cerebral vasculitis, thromboembolism of large arteries, and infectious intracranial aneurysms (Engelen-Lee et al., 2018). Components of the pneumococcal cell wall can be seen weeks after the initial presentation and may be a source of resurging inflammation after the initial immunosuppression by dexamethasone. The demonstrated benefits of adjunctive steroid therapy in reducing pneumococcal meningitis mortality in high-income countries outweigh the possible risks and should continue to be used (Gallegos et al., 2018).

**Table 1:** Studies and Significant Findings on Adjunctive Steroid Therapy for Bacterial Meningitis Treatment by Pathogen.

Reference	Pathogen	N	Primary Findings	Type of Study
de Gans and van de Beek, 2002	*S. pneumoniae*	108	Adults treated with dexamethasone compared to placebo. Unfavorable outcome in 26% of the dexamethasone group and 52% of the placebo group (Relative risk, 0.50; 95 percent confidence interval, 0.30 to 0.83; P=0.006). Dexamethasone associated with a reduction in risk of unfavorable outcomes (relative risk, 0.59; 95 percent confidence interval, 0.37 to 0.94; P=0.03) and reduction in mortality (relative risk of death, 0.48; 95 percent confidence interval, 0.24 to 0.96)	RCT
Brouwer et al., 2010	*S. pneumoniae*	709	Cohort of dexamethasone treatment in 84% of episodes (2006–2009) compared to cohort of dexamethasone treatment in 3% (1998–2002). Rates of death (20% vs. 30%; *p* = 0.001) and hearing loss (12% vs. 22%; *p* = 0.001) were lower in 2006–2009 cohort.	Observational study
Castelblanco et al., 2014	*S. pneumoniae*	21,858	Incidence and inpatient Mortality decreased between 2005 (0·049 per 100 000 people) and 2008 (0·024 per 100 000 people) compared with between 2002 (0·073 per 100 000 people) and 2004 (0·063 per 100 000 people; RR 0·5720, 95% CI 0·4303–0·7582). Temporal association of improved outcomes with recommendations of corticosteroids in clinical practice since 2004	Observational study
Glimåker et al., 2016	*S. pneumoniae*	115	Adults treated with corticosteroids compared to those not given corticosteroids showed lower mortality (10.2% vs. 21.3%, p <0.001). Recovery without sequelae observed increased in corticosteroid-treated compared with non-corticosteroid-treated patients (45.2% vs. 35.1%, p <0.05)	Observational study
Hasbun et al., 2017	*S. pneumoniae*	572	Significantly reduced mortality with the use of adjunctive intravenous steroids. [(6.67% with and 12.5% without steroids, (P=0.0245)].	Observational study
Brouwer et al., 2018	*S. pneumoniae*	1,132	Corticosteroid treatment effects in 25 studies showed mortality reduction from 36% to 29.9%. (RR 0.84, 95% CI 0.72 to 0.98).	Meta analysis
Gallegos et al., 2018	*S. pneumoniae*	120	Adjunctive steroids within 4 hours were more likely given to those with delayed cerebral injury (5/5, 100% vs. 43/115, 37.5%; p=0.01). Adverse effect of delayed cerebral injury found in a higher prevalence (4.1%) in patients with pneumococcal meningitis, associated with adjunctive steroid administration**. **	Observational study
McIntyre et al., 1997	*H. influenzae*	848	Significant reduction in severe hearing loss with dexamethasone treatment in children (combined odds ratio [OR], 0.31; 95% confidence interval [CI], 0.14-0.69).	Meta analysis
Brouwer et al., 2018	*H. influenzae*	825	Corticosteroid treatment of across 25 studies showed significant reduction in the rate of hearing loss overall in children from 12% to 4% after adjunctive corticosteroid therapy. (RR 0.34, 95% CI 0.20 to 0.59) was found.	Meta analysis
Heckenberg et al., 2012	*N. meningitidis*	354	Dexamethasone administered in 17% of patients from 1998-2002 and 90% patients in the 2006-2011 cohort (p<0.001). Rate of arthritis was lower in patients treated with dexamethasone (32 of 258 [12%] vs. 5 of 96 [5%], p = 0.046). Adjunctive dexamethasone not found to improve clinical outcomes significantly.	Observational study
Glimåker et al., 2016	*N. meningitidis*	198	Increased survival and recovery without sequelae in corticosteroid-treated compared with non-corticosteroid-treated patients with N. meningitidis (68.6% vs. 58.1%). Positive trend is observed, but there is no statistically significant change.	Observational study
Glimåker et al., 2016	*Listeria monocytogenes*	77	Adjunctive steroid therapy showed worsened outcome trend compared to non-steroid-treated patients (48.5% vs. 40.0%).	Observational study
Charlier et al., 2017	*Listeria monocytogenes*	818	Higher mortality in patients when given adjunctive dexamethasone (OR 4·58 [1·50-13·98], p=0·008).	Observational study

### *H. influenzae*

The incidence of infection due to *H. influenzae* was reduced significantly in children due to the introduction of the *H. influenzae* type B vaccine. A large 1997 cross-study meta-analysis found a significant reduction in severe hearing loss in *H. influenzae* with dexamethasone treatment in children (McIntyre et al., 1997) (see **Table 1**). More recent data from a meta analysis of corticosteroid use for the treatment of bacterial meningitis found no significant reductions in mortality across 25 studies in *H. influenzae* meningitis cases treated with adjunctive steroid therapy, but similarly did observe a significant reduction in the rate of hearing loss overall in children with *H. influenzae* meningitis from 12% to 4% after adjunctive corticosteroid therapy (Brouwer et al., 2018). Based on the available data, the IDSA guidelines recommend using steroids in children with documented *H. influenzae* meningitis (Tunkel et al., 2004).

### *N. meningitidis*

Vaccination has been available since the 1970s against *N. meningitidis* and used on populations identified as high risk. The incidence of meningococcal meningitis in the US decreased from 1997 to 2010 from 0·721 to 0·123 per 100,000 people (RR 0·1386, 95% CI 0·048–0·4284), which has placed this pathogen close to *H. influenzae* (Castelblanco et al., 2014). Adjunctive dexamethasone does not improve clinical outcomes in meningococcal meningitis but it has been associated with a decrease in arthritis (Heckenberg et al., 2012) (see **Table 1**). In a study from the Swedish quality registry from 1995-2014, there was trend towards decrease mortality with the use of adjunctive steroids in meningococcal meningitis (Glimåker et al., 2016).

### Listeria monocytogenes

*L. monocytogenes* most commonly occurs in neonates, adults above the age of 50 years and in patients with cellular immunodeficiency. In the Swedish study, adjunctive steroids showed a trend towards worse outcomes in patients with Listeria meningitis (48.5% vs. 40.0%) (Glimåker et al., 2016) (see **Table 1**). A large prospective study in France of 818 cases of Listeriosis documented a higher mortality in patients with neurolisteriosis when given adjunctive dexamethasone (OR 4·58 [1·50–13·98], p=0·008) (Charlier et al., 2017). Adjunctive dexamethasone should be discontinued if meningitis is found not to be caused by *S. pneumoniae*, especially if it is caused by *L. monocytogenes* (Hasbun, 2019).

## Encephalitis

Encephalitis is caused by brain tissue inflammation that results in neurological dysfunction from varied etiologies. A nationwide study in the US from 2000–2010, reporting encephalitis hospitalizations, showed unknown etiology in 50% of patients and viral etiologies (48.2%), most commonly herpes simplex virus, *toxoplasma gondii*, and West Nile Virus, with co-morbid HIV present in 7.7% of hospitalizations. Autoimmune encephalitis among other specified causes was reported in 32.5% of cases with known etiology (George et al., 2014). The international consortium of encephalitis diagnostic criteria include 1 major criteria: altered mental status for more than 24 h without alternative diagnosis and several minor criteria: documented fever of >38°C (100.4 F) within 72 h of presentation, seizures not attributed to preexisting seizure disorders, the onset of focal neurological deficits, CSF WBC count >5/cubic mm, new or onset neuroimaging abnormalities consistent with encephalitis presentation, and abnormalities on electroencephalography not due to other causes and consistent with encephalitis presentation (Venkatesan et al., 2013). Possible encephalitis is defined as 1 major criteria with 2 minor criteria; probable encephalitis as 1 major and 3 minor criteria and confirmed if a positive etiology is identified (Encephalitis, Table 3, 2017).

## *Herpes Simplex Virus*

*Herpes simplex virus* (HSV) is the most common cause of sporadic encephalitis and can manifest with temporal lobe abnormalities leading to personality changes, alterations in mental status, decreased consciousness, seizures, and focal neurological deficits (Hasbun et al., 2018). The peak incidence of herpes simplex encephalitis (HSE) occurs in very young children and adults over the age of 50 years with both sexes equally affected and have an incidence of 2–4/1,000,000 persons. HSE is primarily caused by HSV-1, which accounts for 90% of HSE in adults and children, while HSV meningitis is commonly caused by HSV-2 (Bradshaw and Venkatesan, 2016). In about one third of 113 confirmed HSV encephalitis cases, the infection was associated with primary infection of HSV-1. In the remaining cases, the virus was associated with the reactivation of latent virus, demonstrated through serologic assessments (Nahmias et al., 1982). High-dose intravenous acyclovir treatment is recommended for 14–21 days in immunocompetent and immunosuppressed patients, respectively. The benefit of acyclovir treatment has been shown through randomized controlled trials to significantly reduce mortality, establishing it as the standard of care for HSV encephalitis (Bradshaw and Venkatesan, 2016). Furthermore, a delay in starting acyclovir therapy has been associated with worse clinical outcomes (Erdem et al., 2015). The use of adjunctive corticosteroid therapy with acyclovir has shown to be beneficial in mouse and pre-clinical models, showing a reduction in the severity of infection, beneficial long-term effects, and no increase in viral burden (Meyding-Lamade et al., 2003). Despite this evidence, there is very limited clinical data evaluating the use of corticosteroid therapy. Furthermore, there is also the potential concern of potentially increasing viral replication. A small non-randomized retrospective study of 45 patients treated with adjunctive steroids and acyclovir in Japan showed a beneficial impact on clinical outcome and reduction in the extent of HSE infection, without inhibition of the antiviral action of acyclovir (Kamei et al., 2005) (see **Table 2**). The German trial of Acyclovir and Corticosteroids in Herpes-simplex-virus Encephalitis (GACHE) similarly studied the treatment of dexamethasone adjunctive steroid therapy with acyclovir treatment in 41 patients, with 21 patients receiving steroid treatment and 20 receiving a placebo. The small study was stopped prematurely due to slow recruitment and found no significant differences in primary or secondary outcomes between treatment and placebo groups. The primary measured outcome was the functional outcome after 6 months on a modified Rankin scale, and secondary outcomes were the mortality after 6 and 12 months, functional outcomes after 6 and 12 months on the Glasgow outcome scale (GOS), functional outcome after 12 months measured with mRS, quality of life measurements after 6 and 12 months, cranial magnetic resonance imaging findings after 6 months and seizure incidence. This study reinforces the unclear results of corticosteroid adjunctive therapy on HSE and the necessity for larger scale controlled trials (Meyding-Lamadé et al., 2019). Although corticosteroid adjunctive therapy must be studied further as a treatment for HSE, adjunctive corticosteroids are used in practice for patients with increased intracranial pressure and cerebral edema to reduce inflammation.

**Table 2:** Studies on adjunctive steroid therapy for viral encephalitis.

Reference	Diagnosis	N	Primary Findings	Type of Study
Kamei et al., 2005	Herpes Simplex Virus	45	Effects of adjunctive steroids and acyclovir studied. Beneficial impact on clinical outcome and reduction in the extent of HSE infection, without inhibition of the antiviral action of acyclovir.	Observational study
Martinez-Torres et al., 2008	Herpes Simplex Virus	41	Dexamethasone adjunctive steroid therapy with acyclovir versus placebo found no significant differences in primary or secondary outcomes between groups.	RCT
Leis and Sinclair, 2019	West Nile Virus	1	71-year-old woman with weakness, encephalitis, dysphagia and dysarthria, persistent delirium, and stupor improved to wakefulness, after 5-day course of adjunctive steroid treatment.	Case report
Bode et al., 2006	West Nile Virus	228	3/17 patients with corticosteroids died (18%), while 9/48 patients who did not receive adjunctive corticosteroid treatment died (19%).	Observational Study

## West Nile Virus Encephalitis

West Nile virus (WNV) is an arthropod-borne infection that is most commonly transmitted by a mosquito bite. The mosquito injects saliva containing the virus into the host dermis with local viral replication in target cells. The virus then migrates to lymph tissue and then disseminates to the CNS *via* viremia (Colpitts et al., 2012; Hasbun et al., 2018). Incubation periods range from 2 days to 14 days, where 80% of cases remain asymptomatic, 20% have a febrile illness presentation, and 1% present with neuro-invasive disease, which may show aseptic meningitis, encephalitis, or acute flaccid paralysis/myelitis, disrupting the spinal cord (Hasbun et al., 2018). If the virus infiltrates the brain, it commonly infects the basal ganglia, thalamus, and brain stem. Neuronal death, necrosis, and inflammation occur on a microscopic level. Patients presenting with West Nile Encephalitis (WNE) also have chorioretinitis in 50% of cases (Hasbun et al., 2016). Other common symptoms include fever, altered mentation, fatigue, myalgia, headache, stiff neck, rash, vomiting, and diarrhea. Patients diagnosed with WNE have a mortality rate of 15%–18.6%. Currently, no FDA approved treatments vaccine or therapy is approved for WNV infections in humans (Hasbun et al., 2018). Although there are no specific treatments for WNV infections, corticosteroid adjunctive treatment has been used in treatment for and shown rapid improvement effects in a case report of high dose corticosteroid treatment resulting in improvement of a patient from stupor to wakefulness over the course of treatment. The patient was a 71-year-old woman with a WNV infection that rapidly progressed to weakness, encephalitis manifesting with dysphagia and dysarthria, persistent delirium, and stupor. After a 5-day course of steroid treatment she made a rapid improvement to wakefulness. This case study provides the observations of only one patient, so the effect may not be due to the anti-inflammatory steroid treatment (Leis and Sinclair, 2019). A study of 228 patients with WNV in Colorado, found no difference in patients with WNE, 17 patients received corticosteroids and 3 died (18%), whereas 9 of 48 patients who did not receive adjunctive corticosteroid treatment died (19%) (Bode et al., 2006) (see **Table 2**). Other case studies and reviews of small cohorts have shown no significant changes for patients treated with adjunctive steroids with WNV infection (Bakri and Kaiser, 2004). Due to paucity of data, the evidence for the use of corticosteroid therapy in the treatment of WNE is still unclear and must be further investigated in large cohorts.

## Autoimmune Encephalitis

Autoimmune encephalitis is caused by inflammation from the body’s own immune system attacking healthy cells in the brain and spinal cord. Primary autoimmune encephalitis is characterized by immune response directed at cell surface proteins, while paraneoplastic autoimmune encephalitis is a subset associated with tumors and may be the by-product of immune responses to cancerous growth (Hasbun et al., 2017; Hasbun et al., 2018). Autoimmune encephalitis requires clinical criteria of rapid progression in less than 3 months, resulting in neurological debilitation and working memory deficits. Other neurological symptoms include altered level of consciousness, lethargy, personality changes, and other diagnostic findings specific to autoantibodies present in infection. The standard clinical evaluation of patients for autoimmune encephalitis includes clinical presentation, an MRI of the brain, CSF assessment, and EEG. Autoantibodies detected determine disease pathogenesis and classification within autoimmune encephalitis (Graus et al., 2016; Hasbun et al., 2018). The most frequent clinical syndromes are from anti-N-methyl-D-aspartate receptor (NMDAR) encephalitis, characterized by behavioral and psychiatric differences, seizures, memory deficits, and limbic encephalitis, characterized by confusion, agitation, memory deficits, and seizures (Nosadini et al., 2015) (see **Table 3**). Treatment of acute autoimmune encephalitis includes immunotherapy, addressing underlying detected malignancies, and treatment of associated sequelae. Autoimmune encephalitis, characterized by antibodies attacking cell surface proteins, responds to antibody-directed therapy such as intravenous immunoglobulin and plasmapheresis. This treatment is accompanied by the administration of corticosteroids intravenously, such as methyl prednisone. Second-line therapy in the acute phase is rituximab and cyclophosphamide. In the maintenance phase mycophenolate, azathioprine, rituximab, cyclophosphamide, corticosteroids, and intravenous immunoglobulin are administered (Nosadini et al., 2015; Hasbun et al., 2018). Corticosteroids are incorporated into first and second-line immunotherapy for autoimmune encephalitis in conjunction with intravenous immunoglobulin administration. In a review of studies on the treatment of anti-LG1 autoantibody encephalitis, the addition of corticosteroids was shown to have a significant association with the cessation of faciobrachial dystonic seizures (FBDS) in 30% of patients by the first week, and 60% within 2 months. In second-line immune therapy, the most consistent reductions in seizure frequency and mRS score for neurologic disability improvement were associated with steroid treatment (Shin et al., 2013; Nosadini et al., 2015; Shin et al., 2018). The accepted first-line therapies include adjunctive corticosteroid treatment, intravenous immunoglobin, plasma exchange, and immune-adsorption, which have consistently shown positive results in treatment through case studies and systematic review. Corticosteroid therapy in autoimmune encephalitis is used to broadly inhibit the inflammatory response, but possesses little targeted therapy and is associated with systemic side effects, such as the aggravation of psychiatric symptoms like depression, insomnia, agitation, and psychosis, and neurotoxic effects through the potential to induce neuro-degeneration with chronic exposure, making other lines of treatment more effective in combination (Shin et al., 2018). More research must be conducted to determine the optimization of corticosteroid use in conjunction with alternative therapies to maximize clinical benefits and further research on steroid-sparing agents in second-line therapy and long- term maintenance of autoimmune encephalitis.

**Table 3:** Studies on Adjunctive Steroid Therapy for Autoimmune Encephalitis.

Reference	Pathogen	N	Primary Findings	Type of Study
Shin et al., 2013	Autoimmune Encephalitis	14	Patients with LG1 antibodies as a target protein treated with steroids alone were more likely to relapse and had less favorable outcomes than those treated with steroids and intravenous immunoglobins (IVIG).	Case Series
Nosadini et al., 2015	Autoimmune Encephalitis	1,390	Corticosteroids treatment associated with cessation of FBDS (faciobrachial dystonic seizures) within 1 week in 30% (3/10) of patients, and within 2 months in 60% (6/10). mRS improvement consistently associated with corticosteroids second-line immune therapy.	Meta analysis

## Tuberculosis

### Initial Therapy

Tuberculosis (TB) is caused by *Mycobacterium tuberculosis* and in 85% of cases it involves the lungs. One of the most common sites of extra pulmonary TB is the CNS (Hasbun et al., 2018). Tuberculosis meningitis is the most common form of CNS tuberculosis and is found in 10%–15% of children <2 years old after untreated TB infection and is often misdiagnosed as bacterial meningitis (Hasbun et al., 2018). Tuberculosis meningitis is postulated to occur in two stages. First, bacterial lesions form in the brain or meninges, from the dispersion of bacteria through the bloodstream in early infection, leading to the development of meningitis upon entry into the subarachnoid space. Second, proteins and chemicals from the organism leak into the CSF, eliciting an immune response and intense inflammation at the brain and meninges (Hasbun et al., 2018). Bacille Calmette-Guerin (BCG) vaccines can prevent up to 50%–80% of TB meningitis cases, but effectiveness varies with available strains of BCG. The vaccination rates do not meet optimal standards in many areas still (Hasbun et al., 2018). The adjunctive treatment of corticosteroids has been employed to reduce inflammation in tuberculosis meningitis. A randomized, double blind, controlled trial in Vietnam of 545 patients with TB meningitis over 14 years of age with and without human immunodeficiency virus (HIV) infection, studied the use of dexamethasone steroid treatments effects. Treatment with dexamethasone was associated with reduced risk of death and significantly fewer adverse events than in placebo groups, which was consistent across various grades of disease severity and HIV status, providing strong evidence for corticosteroid adjunctive therapy to improve survival in patients over age 14 years with TB meningitis (Thwaites et al., 2004) (see **Table 4**). In a meta analysis of published RCTs of corticosteroid treatment used in TB meningitis, six randomized controlled trials totaling 990 patients were identified, one of which included HIV infected patients. In all studies, the use of corticosteroid adjunctive treatment reduced mortality rates. These results were significant in four of the documented studies. Approximately 39%–73% of the patients across the six trials were severely ill with stage 2 disease (drowsy with focal neurologic deficits) and 23%–56% with stage 3 disease (coma). Faster defervescence, fewer complications of tuberculomas, and fewer clinical complications from anti-tuberculosis medications were documented as well (McGee and Hirschmann, 2008). A Cochrane study has also demonstrated over nine trials and 1,337 participants that use of adjunctive steroids in tuberculosis meningitis reduced deaths by almost one quarter after an 18 month follow up. While the former meta-analysis does not indicate outcome differences between stages of TB infection, the Cochrane study shows that the effect of corticosteroids is significantly consistent across all stages of the disease. Analysis also did not show differences in outcome based on HIV status, although the analysis was noted as underpowered (Prasad et al., 2016). CDC guidelines on the treatment of Tuberculosis based off of published treatment results, recommend the addition of corticosteroids in treatment for tuberculosis meningitis for all patients, although data is limited for patients with HIV (American Thoracic Society, CDC, & Infectious Disease Society of America, 2003; Lewinsohn et al., 2017). Overall, the research shows benefit in reduction of mortality and adverse effects in the use of corticosteroids as an adjunctive treatment in cases of initial therapy for confirmed tuberculosis meningitis.

**Table 4:** Studies and Significant Findings on Adjunctive Steroid Therapy for Tuberculosis Meningitis Treatment.

Reference	Pathogen	N	Primary Findings	Type of Study
Thwaites et al., 2004	*Mycobacterium tuberculosis*	545	Effects of dexamethasone steroid treatments in patients over 14 years of age with and without HIV infection. Dexamethasone treatment was associated with reduced risk of death (relative risk, 0.69; 95 percent confidence interval, 0.52 to 0.92;P=0.01) and significantly fewer adverse events than in placebo groups (26 of 274 patients vs. 45 of 271 patients, P=0.02), consistent across various grades of disease severity and HIV status.	RCT
McGee and Hirschmann, 2008	*Mycobacterium tuberculosis*	990	Across six randomized controlled studies, corticosteroid adjunctive treatment reduced mortality rates and the differences were significant in 4/6 of the trials. Faster defervescence, fewer complications of tuberculomas, and fewer clinical complications from anti-tuberculosis medications documented.	Meta analysis
Murdoch et al., 2007	*Mycobacterium tuberculosis*	-	Adjunctive corticosteroid therapy effective in some cases of TB-IRIS for anti-inflammatory purposes in alleviating symptoms. Corticosteroids effectiveness often anecdotal, requiring larger systematic studies worldwide.	Meta analysis
Meintjes et al., 2010	*Mycobacterium tuberculosis*	110	Patients with TB-IRIS treated with prednisone (1.5mg/kg/day for 2 weeks then 0.75mg/kg/day for 2 weeks) for more rapid improvement in the steroid-treated group arm at 2 weeks (p=0.001) and 4 weeks (p=0.03), and reduced the number of days hospitalized (median cumulative of 0 vs. 3 days; (p=0.009)). Infections occurred in 27 participants in the prednisone arm and 17 in the placebo arm (p=0.05), although the majority of infections were mild.	RCT
Prasad et al., 2016	*Mycobacterium tuberculosis*	1,337	Across nine trials, use of adjunctive steroids in tuberculosis meningitis (with and without HIV) reduced deaths by almost one quarter after an 18-month follow up.(RR 0.75, 95% CI 0.65 to 0.87). Reduction in the risk of death or disabling residual neurological deficit with corticosteroids (RR 0.80, 95% CI 0.72 to 0.89; eight trials, 1314 participants)	Meta analysis

## Paradoxical Reaction

Paradoxical reaction (PR) in tuberculosis is the clinical or radiological worsening of pre-existing TB or the development of new TB lesions in patients who have received treatment against TB and improved in treatment initially. It affects up to 25% of patients and can cause significant morbidity especially in CNS tuberculosis (Bloch et al., 2009). The manifestation of PR in TB patients includes neuroimaging abnormalities, altered CSF pictures, and changes in CSF, including lymphatic pleocytosis, and an increase in CSF protein levels. Corticosteroid treatment has been demonstrated to have beneficial effects through case studies (Teoh et al., 1986; Garcia-Monco et al., 2005; Kim and Kim, 2009; Singh et al., 2016). TB related immune reconstitution inflammatory syndrome (IRIS) can also occur in HIV-positive patients after introducing antiretroviral therapy (ART) and can be life threatening. In a meta-analysis of 8 RCTs it was shown that there was a two-fold increase in TB-IRIS in patients treated with ART. In patients with a new diagnosis of TB and a CD4 count of less than 50/mm^3^, ART within 1–4 weeks of diagnosis improved outcomes. Earlier ART, however, may be associated with more frequent TB-IRIS, so ART therapy is recommended only after 4–6 weeks of diagnosis in patients with HIV-1 TB and higher CD4 counts (Davis et al., 2018). TB related IRIS is more common in adults, occurring in 15.7% of TB patients within 2 months of ART therapy, and is a frequent complication of ART in resource-limited countries, with up to a 30% overall mortality rate (Meintjes et al., 2010; Fane et al., 2018; Hasbun et al., 2018). Adjunctive corticosteroid therapy has shown to be effective in some cases of TB-IRIS for anti-inflammatory purposes in alleviating symptoms. The use of corticosteroids has been variable, however, and effectiveness often anecdotal, requiring larger systematic studies worldwide (Murdoch et al., 2007). A randomized double-blind controlled trial of 110 patients in South Africa shows that patients with TB-IRIS were treated with prednisone resulting in more rapid improvement in the steroid-treated group and reduced the number of days hospitalized with no excess of glucocorticoid adverse drug reaction. Glucocorticoid therapy can be effective in treating TB-IRIS in severe cases, however, the diagnoses of paradoxical TB-IRIS must be correct, and alternate diagnoses must be eliminated completely, due to the existing possibility of adverse effects from steroid adjunctive treatment (Meintjes et al., 2010).

## Cryptococcal Meningitis

### Initial Infection

Cryptococcal meningitis (CM) is an infection of the meninges due to *Cryptococcus neoformans* or *Cryptococcus gatti*, which is less common and thought to cause disease more often in immunocompetent patients (Hasbun et al., 2018). CM is the most prevalent cause of meningitis in populations with high HIV prevalence, particularly as seen in studies conducted in countries and populations with high HIV prevalence in sub-Saharan Africa. It is correlated with a lower CD4 + count in HIV patients (Jarvis et al., 2010; Rajasingham et al., 2015). CM associated with HIV infection causes over 600,000 deaths per year worldwide, with little improvement in treatment. A double-blind randomized controlled trial studied the use of adjunctive dexamethasone steroid therapy in combination with antifungal treatments, amphotericin B and fluconazole, for six weeks in 451 adult patients with CM in Vietnam, Thailand, Indonesia, Laos, Uganda, and Malawi. The trial was stopped prior to completion for safety concerns. A mortality rate of 47% with dexamethasone treatment and 41% in the placebo group at 10 weeks was originally observed, but after 6 months of treatment the corticosteroid treatment group had increased mortality of 57% in comparison to the placebo of 47%. The percentage of patients with disability by 10 weeks of treatment was higher in dexamethasone-treated groups and dexamethasone-treated groups showed more clinically adverse outcomes such as the increased prevalence of grade 3 or 4 infection, renal events, and cardiac events (Beardsley et al., 2016) (see **Table 5**). Based on this study, adjunctive steroids should be avoided in Cryptococcal meningitis.

**Table 5:** Studies and Significant Findings on Adjunctive Steroid Therapy for Fungal and Parasitic CNS Infection.

Reference	Pathogen	N	Primary Findings	Type of Study
Murdoch et al., 2007	*C. neoformans* or *C. gatti*	–	Across small studies and anecdotal benefits, it is reasonable to administer systemic corticosteroids to alleviate unresponsive inflammatory effects.	Meta analysis
Beardsley et al., 2016	*C. neoformans* or *C. gatti*	451	Studied use of adjunctive dexamethasone steroid therapy in combination with antifungal treatments, amphotericin B and fluconazole, for six weeks. Mortality rate of 47% with dexamethasone treatment and 41% in the placebo group (10 weeks) and 57% mortality with dexamethasone treatment in comparison to the placebo of 47% (6 months). Disability and adverse effects higher in dexamethasone-treated groups by 10 weeks of treatment.	RCT
Perfect et al., 2018	*C. neoformans* or *C. gatti*	–	In CM-IRIS major complications, such as CNS inflammation with increased intracranial pressure, corticosteroids (0.5–1.0 mg/kg per day of prednisone equivalent) should be administered and possibly dexamethasone at higher doses for severe CNS signs and symptoms, with a concomitant antifungal regimen.	Meta analysis
Slom et al., 2002	*A. cantonensis*	12	Repeated lumbar punctures and corticosteroid therapy led to improvement of severe headaches and intracranial pressure decrease.	Case series
Maretic et al., 2009	*A. cantonensis*	11	Data supports use of albendazole and mebendazole steroid treatment. Anthelmintic treatment administration not recommended without adjunctive steroid treatment.	Case series
Wang et al., 2012	*A. cantonensis*	–	Small outbreak data supports corticosteroids treatment in combination with anthelmintics	Meta analysis
Pereira-Chioccola et al., 2009	*Toxoplasma gondii*	–	Cases of diffuse encephalitis and expansive lesions with a mass effect in the brain are recommended adjunctive corticosteroid therapy.	Meta analysis
Sonneville et al., 2012	*Toxoplasma gondii*	100	Analyzed patients with HIV and the outcome of adjunctive steroid therapy. With the use of pyrimethamine-sulfadiazine treatment, adjunctive steroids to treat cerebral edema associated with focal lesions are safe but not associated with better neurologic outcomes.	Observational Study
Singhi et al., 2004	*Taenia solium*	133	Disappearance of lesions at 3-month follow up higher (62.9%) in corticosteroid treatment with albendazole group compared to albendazole treatment alone (52.6%). Children in the corticosteroid group had significantly higher seizure recurrence while on AEDs.	RCT
Kishore and Misra, 2007	*Taenia solium*	100	Higher resolution in group that received prednisone alone (68.1%) compared to treatment with antiepileptic monotherapy (60.9%) (p<0.05)	RCT
White et al., 2018	*Taenia solium*	–	Guidelines for the treatment of Neurocysticercosis in America. Corticosteroids should be used in viable parenchymal NCC for reduction in seizure frequency.Corticosteroids should be given with antiparasitic with single enhancing lesion NCC. Corticosteroids should be used, while avoiding antiparasitic treatment with cysticercal encephalitis (with diffuse cerebral edema). Corticosteroids should not be routinely used for calcified parenchymal NCC with or without perilesional edema due to the development of calcifications with perilesional edema in some cases	Meta analysis

### Immune Reconstitution Syndrome (IRIS)

IRIS occurs when immunological recovery after infection contributes to worsening disease in the long term. In HIV patients, after ART and successful immune restoration, inflammatory responses and worsened opportunistic infection has been observed. IRIS is clinically characterized by localized and systemic inflammatory response during invasive fungal infection (Singh and Perfect, 2007). CM-IRIS is seen in HIV patients with CM either as: a) paradoxical IRIS, meaning a relapse of infection after antifungal therapy and initiation of ART or b) as unmasking IRIS, when CM is developed after starting ART and an underlying, previously undiagnosed infection resurfaces. IRIS is developed in 30% HIV and CM co-infected patients who initiate ART. It is increased in individuals with high CSF fungal burdens in initial CM infection and in those who do not clear infection prior to the initiation of antiretroviral treatments (Shelburne et al., 2005; Fane et al., 2018; Hasbun et al., 2018). Systemic corticosteroids have been administered to alleviate inflammation and resulted in positive response through case studies (King et al., 2002; Murdoch et al., 2007). The particular role of adjunctive steroid treatment remains unclear in IRIS, although it has been considered reasonable to administer corticosteroids only in severe cases of unresponsive inflammation (Sharma and Soneja, 2011; Fane et al., 2018).

#### Angiostrongylus cantonensis

*Angiostrongylus* is a parasitic nematode causing severe CNS diseases in humans. *A. cantonensis* from larval invasion causes eosinophilic meningitis as the primary clinical manifestation of the disease. Infection occurs from consuming infected intermediate hosts or vegetables contaminated by these hosts (Martins et al., 2015). Outbreaks in human angiostrongyliasis have been reported in endemic regions such as the Pacific Islands, Southeast Asia, the Caribbean Islands, and Brazil. Treatment includes corticosteroids in combination with anthelmintics (Wang et al., 2012) (see **Table 5**). In a retrospective cohort study, out of 12 hospitalized adults who had traveled to Jamaica and were diagnosed with eosinophilic meningitis, repeated lumbar punctures and corticosteroid therapy led to the improvement of severe headaches in two out of three patients, and intracranial pressure decrease in all three (Slom et al., 2002). A review of case studies demonstrates data favoring the use of antiparasitic (albendazole or mebendazole) and steroid treatment, although anthelmintic administration was not recommended without steroid treatment (Maretic et al., 2009). Anthelmintic treatment is recommended only in early stages of infection, through demonstrated CNS and pulmonary complications when administered beyond 3 weeks of treatment. When anthelmintic treatment is effective later in infection, it kills viable larvae, suspending them in the CNS with negative clinical consequences. Similar effects of delayed resolution of inflammation and disposal of the worm carcass, causing further damage, is noted with prolonged corticosteroid therapy (Prociv and Turner, 2018). Corticosteroid therapy may be beneficial in early treatment, with recommendations to apply the treatment in severe cases, although data is limited in large clinical trials to demonstrate significance and effectiveness in treatment.

#### Toxoplasma gondii

*Toxoplasma gondii*, a single-celled parasite found across the world, causes toxoplasmosis infection. Toxoplasmosis is one of the most common human infections. CNS infection by *T. gondii* causes toxoplasma encephalitis, which is the most common cause of brain mass lesions in HIV infected patients, causing high mortality and morbidity (Vidal, 2019). *T. gondii* causes latent infection in 10%–90% of the world’s population, but clinical presentation otherwise often includes brain abscess and diffuse encephalitis or ventriculitis (Marra, 2018). In patients with HIV, single or multiple ring-enhancing lesions on CT or MRI scans suggest toxoplasma encephalitis in the setting of a positive Toxoplasma IGG. Adjunctive corticosteroid therapy should be considered in patients with brain abscesses with significant cerebral edema (Pereira-Chioccola et al., 2009) (see **Table 5**). The most effective treatment for CNS toxoplasmosis is sulfadiazine and pyrimethamine. Steroid treatment is not recommended unless disproportionate cytotoxic edema is displayed and life threatening. In the case of brain abscess, steroids effectively reduce brain edema, but reduce the effectiveness of host defense processes as well (Muzumdar et al., 2011; Patel and Clifford, 2014). There is no large study demonstrating the efficacy of adjunctive steroid therapy in HIV related toxoplasmosis. Steroid administration is recommended only when lesions due to toxoplasmosis have developed significant mass effect or diffuse brain edema is seen, although the significant benefit of adjunctive steroid therapy in mortality, even in the treatment of cerebral edema, has not been demonstrated in a large cohort (Haverkos, 1987; Rothova et al., 1989; Pereira-Chioccola et al., 2009; Sonneville et al., 2012; Vidal, 2019). Corticosteroids can be used in severe abscess related edema or cases of clinically significant mass effect, with early animal studies showing a reduction in the penetration of antimicrobials into the abscess (Patel and Clifford, 2014).

## Neurocysticercosis

Neurocysticercosis (NCC) is the most common parasitic disease of the CNS in humans, caused by the larval stage of the *Taenia solium* tapeworm. The parasites, once ingested, reach mature size at 3 months, and are established as larval cysts in the tissue, causing single lesions or several lesions in some cases (Garcia et al., 2010). NCC is the single most common cause of acquired epileptic episodes in the developing world, which is the most common presentation of parenchymal NCC (Singhi et al., 2004; Garcia et al., 2010). The Infectious Disease Society of America (IDSA) strongly recommends adjuvant corticosteroid therapy with antiparasitics for anti-inflammatory purposes in patients with viable parenchymal neurocysticercosis (NCC). Steroid therapy has been associated with fewer seizures, although optimal doses have not yet been defined. For single enhancing lesions due to NCC, corticosteroids concomitant with antiparasitic treatment is also strongly recommended, due to data on worsening symptoms with antiparasitic treatment alone (Singhi et al., 2004; White et al., 2018). In the case of calcified parenchymal NCC with or without perilesional edema, corticosteroid therapy is not routinely recommended. In cysticercal encephalitis with diffuse cerebral edema, the IDSA recommends that corticosteroid treatment is administered, without the use of antiparasitic drugs, because antiparasitic treatment is associated with worsening edema. The guidelines show that evidence from large case series and overall evaluation points to antiparasitic treatment with steroid therapy, over antiparasitic treatment alone, as antiparasitic drugs have been shown to worsen the symptoms of NCC (White et al., 2018). Corticosteroids are the primary treatment for chronic cysticercosis arachnoiditis or encephalitis NCC (Garcia et al., 2010). Due to the varying nature of antiparasitic treatment in combination with adjunctive corticosteroid therapy across different presentations of NCC, treatment of NCC with must be individualized (Singhi et al., 2004).

## Bacterial Brain Abscess

Brain abscess is a focal infection of the brain with localized inflammation of the cerebrum. Bacteria is responsible for over 95% of all brain abscesses, entering the brain through the contiguous spread, hematogenous dissemination, or as a consequence of distant infectious foci in other organs. (Patel and Clifford, 2014; Sonneville et al., 2017). The incidence of bacterial brain abscess is around 0.3–0.9/100,000 inhabitants per year in developed countries (Sonneville et al., 2017). Clinical symptoms of bacterial brain abscess include fever, focal neurological deficits, seizures, and altered consciousness. In addition to antibacterial treatment that is recommended upon identification of the condition, patients with warning signs of brain herniation can be placed on a short course of adjunctive steroids (Sonneville et al., 2017). Steroid administration is not typical to bacterial brain abscess and is not recommended unless life-threatening issues of cytotoxic edema are present and have led to clinical mass effect (Miranda et al., 2013). In these cases, the adjunctive corticosteroid therapy may be effective in reducing antimicrobial penetration into the abscess. Although steroid therapy is effective due to anti-inflammatory properties in the case of brain edema, the host’s defense mechanisms may be disrupted. Steroids have also been shown to inhibit collagen capsule formation, which may inhibit a key mechanism for leucocyte travel in the body’s defense systems (Muzumdar et al., 2011; Patel and Clifford, 2014) (see **Table 6**).

**Table 6:** Studies on adjunctive steroids use for the treatment of bacterial brain abscess.

Reference	Pathogen	N	Primary Findings	Type of Study
Muzumdar et al., 2011	Brain Abscess	289	Steroid administration is not recommended unless life-threatening issues of cytotoxic edema. Intravenous were steroids administered for 15 patients, who presented with significant perifocal edema. Steroid therapy was tapered over 2 weeks and edema was markedly reduced with resolution of the abscess cavity after steroid therapy.	Observational Study
Patel and Clifford, 2014	Bacterial Brain Abscess	–	Steroid administration is not recommended unless severe abscess related edema has led to clinically significant mass effect. Corticosteroid therapy may reduce antimicrobial penetration into the abscess.	Meta analysis

## Conclusion

Adjunctive steroids therapy has proven effective to improved outcomes in some cases of CNS infection, although in others administration of steroid therapy can be detrimental. Critical evaluation of the presenting infection and point of administration is necessary for optimal therapeutic use as indicated through the review. Adjunctive steroids are effective in reducing inflammation and improving clinical outcomes in some causes of meningitis such as *S. pneumoniae* (mortality), *H. influenzae* (hearing loss), *N. meningitidis* (arthritis), and *M. tuberculosis* (mortality). Even though the use of adjunctive steroids is an essential therapy of autoimmune encephalitis, its use in viral encephalitis is unclear as there are limited studies. Steroids are also used in *A. cantonensis* meningitis and in brain abscesses caused by bacteria or by toxoplasmosis when associated with significant mass effect or cerebral edema. Adjunctive corticosteroid therapy is also consistently recommended in neurocysticercosis with cerebral edema. Adjunctive corticosteroids are also used in IRIS treatment to alleviate inflammation in severe cases. The use of corticosteroid therapy can also be detrimental as in *L. monocytogenes* or CM so an accurate, prompt diagnosis is key. Overall, data for the use adjunctive steroids in several CNS infections (e.g., HSV, WNV, etc.) are limited and future studies should explore the utility of steroids in larger-scale studies.

## Author Contributions

RH conceived the general idea and provided critical revision and final approval of the manuscript. SG conducted the literature review and wrote the draft manuscript. All authors contributed to the article and approved the submitted version.

## Funding

The Grant A Starr Foundation supported this work.

## Conflict of Interest

RH has received research support and personal fees from Biofire.

The remaining authors declare that the research was conducted in the absence of any commercial or financial relationships that could be construed as a potential conflict of interest.
